# Supplementary material for: Carrier transport theory for twisted bilayer graphene in the metallic regime
Source: Nat Commun. 2021 Sep 30;12:5737. doi: 10.1038/s41467-021-25864-1 (PMC8484653; doi:10.1038/s41467-021-25864-1)
Supplement: Supplementary file 1 — Supplementary Information [file 41467_2021_25864_MOESM1_ESM.pdf]

Supplementary Information for Carrier transport theory for twisted bilayer graphene in the metallic regime

Gargee Sharma,[1, 2, *] Indra Yudhistira,[1, 3, *] Nilotpal Chakraborty,[1, 4, 5] Derek Y. H. Ho,[1, 4] M. M. Al Ezzi,[1, 3] Michael S. Fuhrer,[6, 7] Giovanni Vignale,[1, 4, 8] and Shaffique Adam[1, 3, 4, 9, †]

[1]*Centre for Advanced 2D Materials, National University of Singapore, 6 Science Drive 2, 117546, Singapore*
[2]*School of Basic Sciences, Indian Institute of Technology Mandi, Mandi-175005, India*
[3]*Department of Physics, National University of Singapore, 2 Science Drive 3, 117551, Singapore*
[4]*Yale-NUS College, 16 College Avenue West, 138527, Singapore*
[5]*Rudolf Peierls Centre for Theoretical Physics, Clarendon Laboratory, Parks Road, Oxford OX1 3PU, UK*
[6]*ARC Centre of Excellence in Future Low Energy Electronic Technologies, Monash University, Monash, Victoria 3800, Australia*
[7]*School of Physics and Astronomy, Monash University, Monash, Victoria 3800, Australia*
[8]*Department of Physics and Astronomy, University of Missouri, Columbia, Missouri 65211, USA*
[9]*Department of Materials Science and Engineering, National University of Singapore, 9 Engineering Drive 1, 117575, Singapore*

I. INTERPLAY BETWEEN INTER-BAND AND INTRA-BAND SCATTERING

The Dirac approximation of tBG consists of a degenerate Dirac cone with a renormalized Fermi velocity v_F which is heavily suppressed near the magic angle θ_M. Even though this approximation is valid only below the Van Hove singularity (VHS), it nevertheless allows us to analytically examine the interesting qualitative features in the electronic transport of tBG. We show that the renormalization of the Fermi velocity has important implications on phonon assisted scattering mechanisms particularly in the regime when the Fermi velocity becomes close to the sound velocity, a situation which is rather unusual in typical metals. Since both θ_M and $\theta_{\rm cr}$ are very close, the magic angle physics becomes even more interesting with the interplay of electrons and phonons, which gives specific signatures in the electronic transport. While v_F can be controlled by twist angle, $c_{\rm ph}$ is relatively insensitive to changes in the twist angle.

From the best estimates available for LA phonons in graphene, bilayer graphene, twisted bilayer graphene [1] and graphite, we expect the effective phonon velocity $c_{\rm ph}$ defined by $2/c_{\rm ph}^2 = 1/c_{\rm LA}^2 + 1/c_{\rm TA}^2$ in the current scenario to lie in the range of $c_{\rm ph} \sim$ 20-30 km s^{-1}, and not heavily sensitive to changes in the twist angles (see Supplementary Table I). It has also been shown that the tBG phonon spectrum is largely insensitive to the details of moiré superlattice even at small twist angles close to 1° [2].

Supplementary Table I. Comparison of phonon sound velocity in various Carbon based materials.

System	Sound velocity (km/s)
monolayer graphene	∼ 20 [3–5]
bilayer graphene	∼ 20 [1]
graphite	∼ 21 [6]
carbon nanotubes	∼ 21 [7]
twisted bilayer graphene	∼ 20 [1]

We now discuss the kinematics of electron-phonon scattering focusing on small values of the renormalized Fermi velocity. Specifically two types of processes are possible. An electron in band λ with momentum $\mathbf{k}$ can scatter into an electron in band λ' with momentum $\mathbf{k} \pm \mathbf{q}$ due to emission or absorption of a phonon. The case $\lambda = \lambda'$ ($\lambda \neq \lambda'$) correspond to intra (inter) band scattering respectively. Additionally we also have the requirement of energy conservation $\varepsilon_{\mathbf{k}\pm\mathbf{q},\lambda'} = \varepsilon_{\mathbf{k},\lambda} \pm \hbar c_\nu q$. We can visualize the conditions for intraband and interband phonon scattering process diagrammatically as shown in Supplementary Fig. 1. If $\mathbf{k}_{\rm in}$ is the initial wave vector, and $\mathbf{q}$ is the phonon wavevector, then $k_{\rm in} - (c_{\rm ph}/v_F)q$ denotes the locus of allowed final states as dictated by conservation of energy for

* These authors contributed equally: Gargee Sharma, Indra Yudhistira
† Corresponding author; shaffique.adam@yale-nus.edu.sg

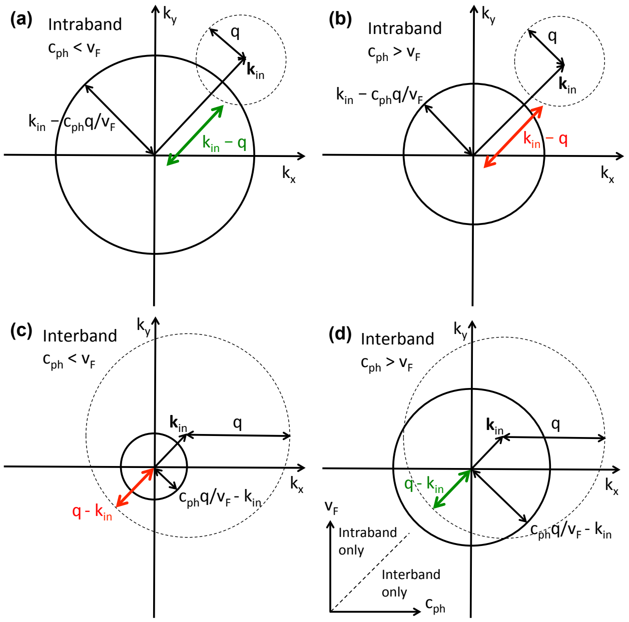

Supplementary Figure 1. Kinematic constraints imply that only intraband phonon scattering is allowed for Fermi velocity larger than the phonon velocity ($v_F > c_{\rm ph}$) and only interband scattering is allowed for $v_F < c_{\rm ph}$. The solid (dashed) circle displays the locus of final electronic momenta allowed by conservation of energy (momentum). Intraband electronic transitions are kinematically allowed when (a) $c_{\rm ph} < v_F$ and forbidden when (b) $c_{\rm ph} > v_F$. On the other hand, interband electronic transitions are kinematically forbidden when (c) $c_{\rm ph} < v_F$ and allowed when (d) $c_{\rm ph} > v_F$. In all panels, $k_{\rm in}$ refers to the initial electronic wave vector and q to the phonon wave vector. Inset: Depending on the relationship between $c_{\rm ph}$ and v_F, either intraband or interband transitions are allowed. Similar statements can be proven for phonon absorption using similar kinematic diagrams. In twisted bilayer graphene, Fermi velocity decreases with twist angle, and we define the critical angle $\theta_{\rm cr}$ where $v_F = c_{\rm ph}$. Here, the electron-phonon scattering length diverges resulting in several orders of magnitude decrease in the resistivity. As discussed in the main text, we estimate $\theta_{\rm cr} \approx 1.15^\circ$ which is always larger than the magic angle defined here as when $v_{\rm F}$ vanishes.

intraband scattering, while $(c_{\rm ph}/v_F)q - k_F$ is the locus of final allowed states for interband scattering, indicated by a solid circle. The locus of the final states dictated by momentum conservation is indicated by the dotted circle should intersect with the solid circle for scattering to take place. Based on this observation we can conclude that an intraband phonon scattering process is kinematically forbidden if $c_{\rm ph} > v_F$, while interband scattering is forbidden if $c_{\rm ph} < v_F$. As one approaches magic angle, one necessarily crosses over from intraband to the intraband regime.

The above kinematics has implications on the electron-phonon scattering rate. The electron-phonon scattering time for the Dirac model of tBG can be solved within the Boltzmann formalism (see Sec. IV for calculation details). In the limit $v_F \to c_{\rm ph}$ (i.e. at $\theta \to \theta_{\rm cr}$) we specifically evaluate from Supplementary Eq. 17 that $1/\tau^{e-{\rm ph}}_{\rm inter}(\varepsilon, T) \to 0$ as well as $1/\tau^{e-{\rm ph}}_{\rm intra}(\varepsilon, T) \to 0$, highlighting that the scattering phase space exactly vanishes at this critical point. The electron-phonon scattering time can be further simplified deep in the intraband regime ($v_F \gg c_{\rm ph}$) and large-T ($k_B T \gg \hbar\omega_{\mathbf{q}}$) to be

$$\frac{1}{\tau^{e-{\rm ph}}_{\rm intra}(\varepsilon, T)} = \sum_{\nu={\rm LA,TA}} \frac{1}{4}\left(\frac{|\varepsilon|\zeta(\theta)^2}{\hbar^2\mu_s}\right)\left(\frac{1}{c_\nu^2 v_F^2}\right)\left(\frac{k_B T}{\hbar}\right). \tag{1}$$

The scattering rate is $T-$linear and density independent at high-T, consistent with earlier findings in the literature [8].

The interband regime, which is of interest especially in the vicinity of the magic angle remains unexplored so far in the literature. The electron-phonon scattering rate can also be simplified deep in the interband regime ($v_F \ll c_{\rm ph}$) and at high-T ($k_B T \gg \hbar\omega_{\mathbf{q}}$) to be (see Sec. IV)

$$\frac{1}{\tau^{e-{\rm ph}}_{\rm inter}(\varepsilon, T)} = \sum_{\nu={\rm LA,TA}} \left(\frac{|\varepsilon|\zeta(\theta)^2}{\hbar^2\mu_s}\right)\left(\frac{v_F^2}{c_\nu^6}\right)\left(\frac{k_B T}{\hbar}\right). \tag{2}$$

Supplementary Fig. 2 plots the interband and intraband scattering rates as a function of energy. The numerical results closely agree with the above analytical expressions at high-T. The usual intraband scattering is linear in temperature for $T > T_{\rm BG}/4$ and $E \ll k_B T$. Further, we find that the interband scattering is also linear in temperature, but for $T > T_F/2$. This explains how phonon scattering can give linear-in-T behavior at temperatures well below $T_{\rm BG}$.

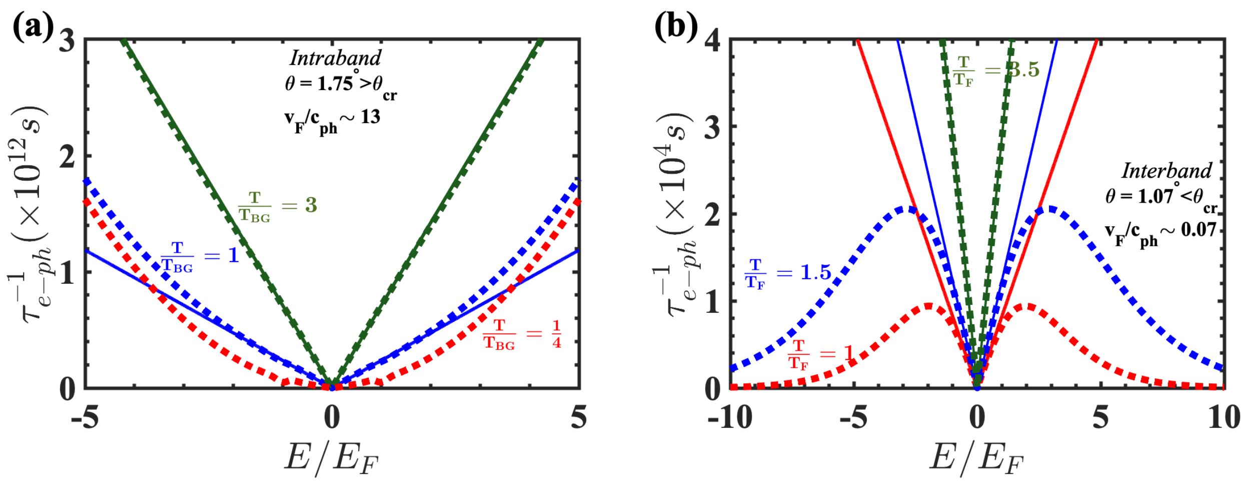

Supplementary Figure 2. Interband and intraband phonon scattering are qualitatively different. Data points are a numerical solution of Supplementary Eq. 17. (a) The usual intraband scattering is linear in temperature for $T > T_{\rm BG}/4$ and $E \ll k_B T$, (solid lines are the analytical expression, Supplementary Eq. 1). (b) We find that the interband scattering is also linear in temperature, but for $T > T_F/2$. This explains how phonon scattering can give linear-in-T behavior at temperatures well below $T_{\rm BG}$. Solid lines are the analytical result, Supplementary Eq. 2. We note that while intraband scattering becomes stronger for $E \gg k_B T$, interband scattering becomes weaker in the same limit.

II. DYNAMICAL SCREENING OF ELECTRON-PHONON COUPLING

The role of screening is crucial in a phonon dominated carrier scattering theory, and as we shall see it is even more non-trivial in the case of tBG. Within a simple Thomas-Fermi screening model, we qualitatively note that the divergence of the dielectric constant $\epsilon^{-1} \sim v_F$ for vanishing Fermi velocities is exactly canceled by the the v_F^{-1} dependence of resistivity for most scattering mechanisms including the scalar phonon modes [9]. Gauge phonons on the other hand are unaffected by screening, which dramatically enhances its importance close to the magic angle (see Sec. III for more details on gauge and scalar phonon modes in tBG). Particularly, the crossover temperature for which gauge phonons dominate over charged impurities drops from room temperature in monolayer graphene to the order of few Kelvins in tBG close to the magic angle. This conclusion holds even when we consider static screening calculated within the random phase approximation (RPA) i.e. $\epsilon(\mathbf{q}, \omega \to 0)$. The effect of frequency dependence of $\epsilon(\mathbf{q}, \omega)$ on screening of the electron-phonon vertex has not been considered so far. This is because typically static screening is a good approximation in metals when screening due to impurities or phonons is considered. However, the frequency dependent dielectric function $\epsilon(\mathbf{q}, \omega)$ is essential to describe properties like the dynamic screening, which becomes essential in tBG because electronic and phononic energy scales are quantitatively very similar. Recently we have discussed the role of $\epsilon(\mathbf{q}, \omega)$ on superconductivity in tBG [10]. Here we show the dynamic screening properties of scalar deformation potential phonon modes within the RPA.

The basic building block of RPA screening is the polarizability bubble $\Pi_C(\mathbf{q}, \omega)$, which can be written in the most general form as

$$\Pi_C(\mathbf{q}, i\omega_m) = \frac{1}{\beta}\frac{g}{A}\sum_{\mathbf{k}, i\omega_n} \mathrm{Tr}[G(\mathbf{k}, i\omega_n) G(\mathbf{k}+\mathbf{q}, i\omega_n + i\omega_m)], \tag{3}$$

where the summation $i\omega_n$ is over the imaginary frequency, g is the degeneracy in tBG, $G(\mathbf{k}, i\omega)$ is the Green's function, and the trace is over the sublattice degrees of freedom. Performing the trace and the Matsubara summation we obtain

$$\Pi_C(\mathbf{q}, i\omega) = \lim_{\eta \to 0^+} \frac{g}{A} \sum_{\lambda, \lambda', \mathbf{k}} \frac{f^0_{\mathbf{k},\lambda} - f^0_{\mathbf{k}+\mathbf{q},\lambda'}}{\hbar\omega + \varepsilon_{\mathbf{k},\lambda} - \varepsilon_{\mathbf{k}+\mathbf{q},\lambda'} + i\eta} F^{\lambda\lambda'}_{\mathbf{k},\mathbf{k}+\mathbf{q}} \tag{4}$$

where $F^{\lambda\lambda'}_{\mathbf{k},\mathbf{k}+\mathbf{q}} = (1 + \lambda\lambda' \cos\theta_{\mathbf{k},\mathbf{k}+\mathbf{q}})/2$ is the tBG chirality factor within Dirac model and the equilibrium Fermi distribution function is $f^0_{\mathbf{k},\lambda} = [\exp\{(\varepsilon_{\mathbf{k},\lambda} - \mu)/(k_B T)\} + 1]^{-1}$. The dielectric function $\epsilon(\mathbf{q}, i\omega_m)$ is given by the RPA summation and is related to the basic pair bubble as $\epsilon(\mathbf{q}, \omega) = 1 - V_{\mathbf{q}}\Pi_C(\mathbf{q}, i\omega)$, where $V_{\mathbf{q}} = 2\pi e^2/\kappa q$ is the Fourier transform of the Coulomb potential, κ being the dielectric constant. We evaluate Π_C and $\epsilon(\mathbf{q}, \omega)$ for finite frequencies

and temperatures, semi-analytically. To the best of our knowledge, this has not been done previously in the literature, at least in the context of phonons. The electron-phonon vertex is renormalized as $g_{\mathbf{q}} \to g_{\mathbf{q}}/\epsilon(\mathbf{q},\omega)$. In Supplementary Fig. 3a we plot the dynamic dielectric constant $|\epsilon(q,\omega)|$ for monolayer graphene and tBG at small twist angles, and discuss its implications on screening of the electron-phonon vertex $g_{\mathbf{q}}$. The static screening case (long wavelength limit) corresponds to the limit of $\omega \to 0$, where $\epsilon(\mathbf{q})$ is always real and greater than unity. Therefore the electron-phonon coupling is always screened compared to the bare value as expected. Dynamic screening, on the other hand, must be considered separately in the interband ($v_F < c_{ph}$) and intraband regimes ($v_F > c_{ph}$). In the intraband regime, the phonon spectrum always lies in the region when $|\epsilon(q,\omega)| > 1$, and therefore the vertex $g_{\mathbf{q}}$ in this case will be always screened. In the interband regime, the phonon spectrum intersects regions where $|\epsilon(q,\omega)|$ can be both greater or smaller than one. This is because the finite frequency dielectric function can become imaginary with its absolute value $|\epsilon(\mathbf{q},\omega)|$ becoming less than one for certain values of $\mathbf{q}$ and $\omega > v_F q$. When $|\epsilon(q,\omega)| < 1$, the vertex $g_{\mathbf{q}}$ is in fact anti-screened, in which case the electron-phonon coupling will be enhanced rather than suppressed. For the interband regime, the effect on the scattering rates and resistivity need to be explicitly calculated (since these involve an integral over all possible wavevectors). We find that the for intraband scattering, the dynamic screening and static screening give results that are qualitatively and quantitatively similar; however, for interband scattering, the full dynamical screening is necessary. Another feature which is evident from Supplementary Fig. 3b is that the phase space when $|\epsilon(q,\omega)| < 1$ is reduced in tBG due to the suppressed Fermi velocity. This can be understood qualitatively by specifically searching for the zeros of the dielectric function ($|\epsilon(q,\omega)| = 0$), which gives us the plasmon spectrum ω_{pl}. The low-q limit of the plasmon spectrum $\omega_{pl} = \sqrt{ge^2 E_F q/2\kappa}$. Clearly, $q/k_F = (2\kappa v_F/ge^2)(\omega_{pl}/E_F)^2$. Therefore if we fix ω/E_F, the dimensionless wavevector q/k_F decreases with decrease of v_F. This argument can be extended to the general case when $|\epsilon(q,\omega)| < 1$, and the shrinking of blue region in Supplementary Fig. 3 is expected. Supplementary Fig. 4 plots $|\epsilon(q,\omega)|$ for graphene and tBG (within the Dirac model) at different temperatures and frequencies. Again, the suppression of anti-screening and enhancement of screening effects in tBG is highlighted.

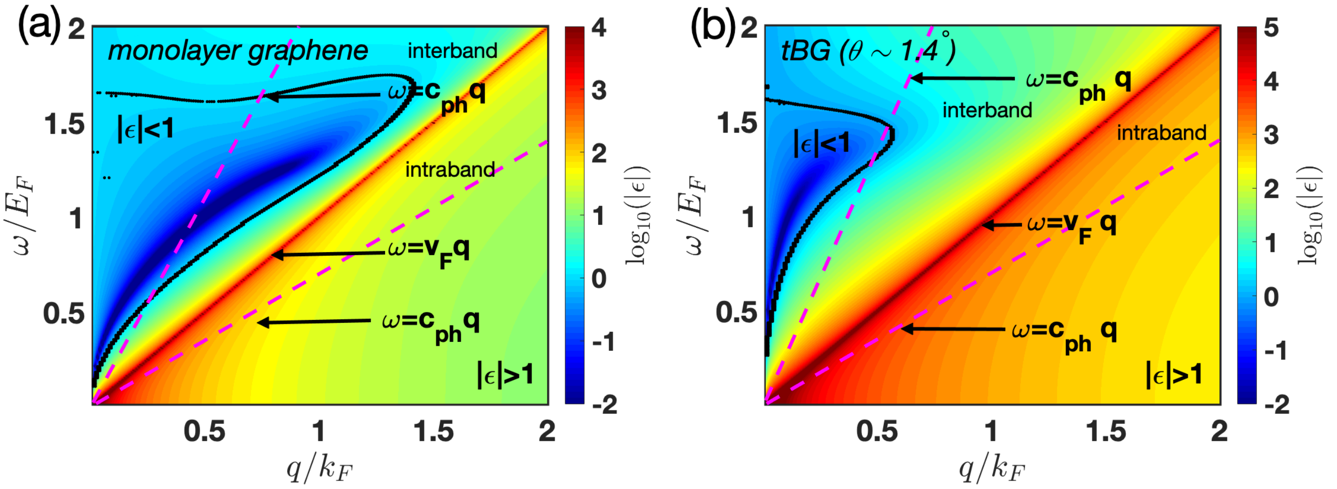

Supplementary Figure 3. The finite temperature dynamical dielectric function $|\epsilon(q,\omega)|$ in (a) monolayer graphene and (b) tBG at $\theta_M = 1.4^\circ$ within the Dirac approximation, plotted as a function of q/k_F and ω/E_F for $T = 10$K, $\kappa = 4$, and $n = 10^{11}\text{cm}^{-2}$, in the $\log_{10}$ scale. The regions where $|\epsilon(q,\omega)| > 1$ and $|\epsilon(q,\omega)| < 1$ are separated by the black curve. The phonon spectrum ($\omega = c_{\text{ph}} q$) for the intraband process ($c_{\text{ph}} < v_F$) is indicated by the dashed lines, while the interband process is indicated by the dotted lines. Notice that for the interband phonons, the spectrum may intersect regions where the dielectric function becomes less than 1, leading to anti-screening of the electron-phonon vertex at those points. However, this anti-screening region shrinks in tBG due to the reduced Fermi velocity and makes negligible contribution to the electron-phonon scattering rate.

One might expect that when the plasmon dispersion becomes exactly equal to the phonon dispersion, there will be a divergent contribution leading to overall anti-screening. However it is not the case. The value of the dimensionless wavevector q/k_F where the two dispersions intersect (other than the trivial point $q = 0$) is given by $q/k_F = ge^2 v_F/2\kappa\hbar c_{\text{ph}}^2$. The intersection point decreases at smaller values of v_F. Plugging in typical values ($\kappa \sim 4$, $c_{\text{ph}} \sim 20000$ m/s), and very close to the magic angle ($\theta \sim 1.07^\circ$), we find $q/k_F \sim 25$, which is way beyond the interband kinematics regime (As Supplementary Eq. 28 suggests that the scattering wavevector $q/k \ll 1$ in the kinematically allowed interband regime when $v_F \ll c_{\text{ph}}$). Thus the possibility of an overall anti-screening is ruled out. In fact, if anti-screening was dominant, it could likely explain the large observed values of deformation potential extracted from the experiments [11]. We also explicitly calculate the net effect of screening and anti-screening on the scattering rates.

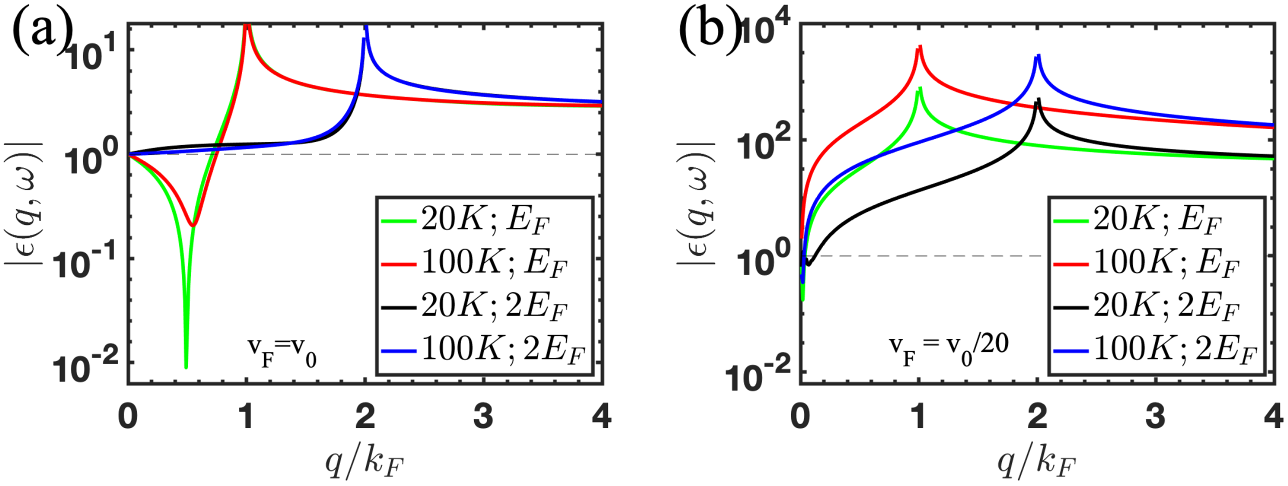

Supplementary Figure 4. $|\epsilon(q,\omega)|$ for graphene (a) and tBG (b) at different temperatures and frequencies. The suppression of anti-screening and enhancement of screening effects in tBG is observed. Here v_0 is the Fermi velocity in monolayer graphene.

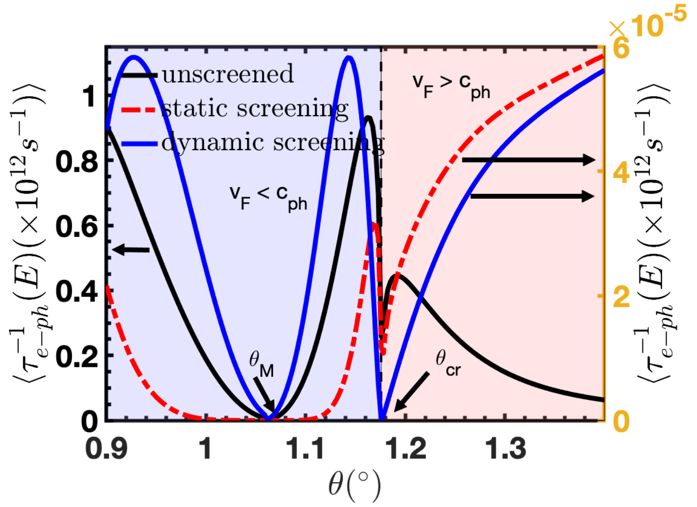

Supplementary Figure 5. A comparison of energy averaged electron-phonon scattering rates (unscreened vs. static screening vs. dynamic screening) in tBG as a function of twist angle for $T = 10$K, $n = 5 \times 10^{10}\text{cm}^{-2}$. The shaded red (blue) regions indicate regions when $v_F > c_{\text{ph}}$ ($v_F < c_{\text{ph}}$) and the dip occurs at θ_{cr} when $v_F = c_{\text{ph}}$. The unscreened scattering rate is at least three orders of magnitude larger than the screened version. Further, static screening is qualitatively inaccurate in the interband scattering regime ($c_{\text{ph}} > v_F$).

We compare the electron-phonon scattering rates for unscreened, static screening, and dynamic screening in tBG as a function of twist angle focusing on the regime close to the magic angle and the critical angle (see Supplementary Fig. 5). Static screening is a good approximation for $\theta > \theta_{\text{cr}}$ though it could be quantitatively inaccurate close to θ_{cr}. However static screening is qualitatively inaccurate when $\theta_M < \theta < \theta_{\text{cr}}$. Therefore, in order to correctly describe screening properties of scalar phonon modes one must take into account the full frequency dependent dynamical dielectric function. Nevertheless, the unscreened scattering rate is at least three orders of magnitude higher than the dynamically screened scattering rate, even when $\theta_M < \theta < \theta_{\text{cr}}$. The anti-screening of vertex for few values of momentum q is compensated by the large screening at other values of q. The overall screening, and not antiscreening of the electron-phonon coupling, is attributed to the shrinking of the $|\epsilon(q,\omega)|$ phase space in Supplementary Fig. 3 for small values of v_F. The comparison of resistivities also shows the same pattern as the scattering rates. This explicitly shows that only the gauge phonon modes are relevant and the scalar deformation potential is irrelevant.

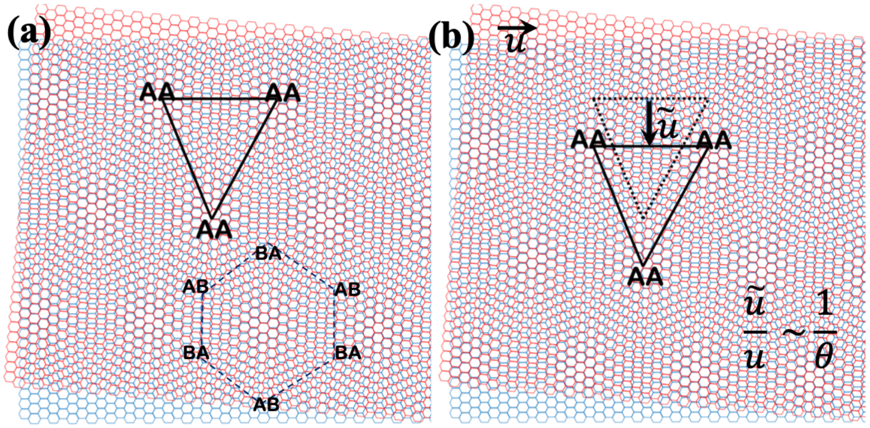

Supplementary Figure 6. Graphical illustration of the enhancement of antisymmetric gauge phonon mode ($\tilde{\beta}_A$) in tBG. A small displacement u in one layer causes an enhanced displacement $\tilde{u} \sim 1/\theta$ in the moiré superlattice. (a) superlattice of AA sites (solid lines), and also AB-BA sites (dashed lines) in twisted bilayer graphene, (b) a tiny displacement in the x direction (indicated by horizontal arrow) of the top layer (red) results in a large displacement of the AA sites in the y direction (indicated by vertical arrow), while preserving the area of the superlattice. The dotted and the solid black lines indicate undisplaced and displaced lattice respectively.

III. ENHANCEMENT OF ANTISYMMETRIC GAUGE PHONON MODE

When two layers of graphene are stacked on top of each other, there are four in-plane interlayer acoustic modes (LA1, LA2, TA1, TA2) [1]. Two of these modes are layer symmetric, i.e both layers move in the same direction and the other two are layer anti-symmetric. In this section we calculate the effect of these modes on the electron-phonon coupling of the superlattice. We can map the antisymmetric modes of tBG on to an effective monolayer by considering the superlattice of AA stacking centers, which form a triangular lattice (see Supplementary Fig. 6). The AB and BA stacking centers form a dual hexagon superlattice (Supplementary Fig. 6). Since within the Dirac model we treat the superlattice as graphene with a renormalized Fermi velocity we can express the electron-phonon coupling matrix for the superlattice as [5, 7] :

$$H_{\text{e-ph}} = \begin{pmatrix} D'_A(\tilde{u}_{xx} + \tilde{u}_{yy}) & \beta'_A(\tilde{u}_{xx} - \tilde{u}_{yy} - i(\tilde{u}_{xy} + \tilde{u}_{yx})) \\ \beta'_A(\tilde{u}_{xx} - \tilde{u}_{yy} + i(\tilde{u}_{xy} + \tilde{u}_{yx})) & D'_A(\tilde{u}_{xx} + \tilde{u}_{yy}) \end{pmatrix} \tag{5}$$

where D'_A and β'_A are the deformation potential constant and the gauge field coupling constant of the superlattice respectively. We can write $\beta'_A = \beta_A v_F/v_0$, where β_A is the coupling constant for monolayer graphene, since the gauge-field coupling constant is proportional to the Fermi velocity [5]. This factor causes a suppression since there is a reduction in Fermi velocity in tBG near magic angle. $\tilde{u}_{ij} = \partial\tilde{u}_i/\partial j$ and $\tilde{\mathbf{u}}$ is the displacement of the AA/AB sites of the superlattice. Let us first consider the anti-symmetric modes. A relative displacement of $\mathbf{u}$ between the two layers causes the AA sites of the superlattice to be displaced by $|\tilde{\mathbf{u}}| = \gamma|\mathbf{u}|$ in the perpendicular direction [12], where $\gamma = 1/[2\tan(\theta/2)]$. Hence we get:

$$\begin{aligned} \tilde{u}_{xx} &= \gamma u_{yx}, \quad \tilde{u}_{yy} = -\gamma u_{xy} \\ \tilde{u}_{xy} &= \gamma u_{yy}, \quad \tilde{u}_{yx} = -\gamma u_{xx} \end{aligned} \tag{6}$$

On substituting these results into Supplementary Eq. 5 and using the symmetric property of the in plane strain tensor ($u_{xy} = u_{yx}$) we can write and effective electron-phonon coupling Hamiltonian in terms of the monolayer strain tensor as

$$H^A_{\text{e-ph}} = \begin{pmatrix} 0 & \tilde{\beta}_A(2u_{xy} + i(u_{xx} - u_{yy})) \\ \tilde{\beta}_A(2u_{xy} - i(u_{xx} - u_{yx})) & 0 \end{pmatrix} \tag{7}$$

where the superscript A indicates the antisymmetric phonon mode contribution, $\tilde{\beta}_A = \gamma\beta_A(v_F/v_0)$, $\tilde{D}_A = 0$ are the effective gauge field coupling constant and the effective deformation potential constant respectively. Hence we see

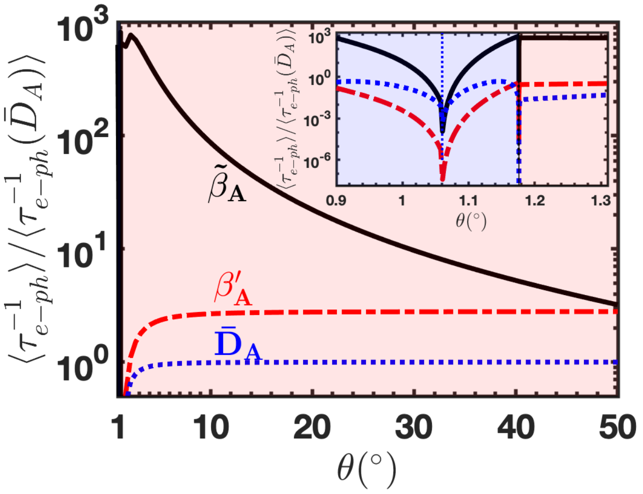

Supplementary Figure 7. Comparison of electron-phonon scattering rates for the antisymmetric gauge mode ($\tilde{\beta}_A$), symmetric gauge mode (β'_A), and the dynamically screened scalar (deformation) potential ($\bar{D}_A$). The inset shows the behaviour near magic angle. The scattering rates are normalized with respect to the screened monolayer deformation value $\bar{D}_A$. The two gauge phonon modes $\tilde{\beta}_A$ and β'_A are comparable to each other at large angles; however, near θ_M, $\tilde{\beta}_A$ dominates by several orders of magnitude.

that antisymmetric phonon modes cause a large enhancement in the electron-gauge phonon coupling at low angles which counters the reduction in Fermi velocity. We also find that there is no scalar field (diagonal) contribution from the antisymmetric modes as expected because these modes are area preserving. The symmetric phonon modes do not have a similar enhancement and they resemble the usual acoustic phonon contribution in graphene [12, 13], hence

$$H^S_{\text{e-ph}} = \begin{pmatrix} D'_A(u_{xx}+u_{yy}) & \beta'_A(u_{xx}-u_{yy}-2iu_{xy}) \\ \beta'_A(u_{xx}-u_{yy}+2iu_{xy}) & D'_A(u_{xx}+u_{yy}) \end{pmatrix} \tag{8}$$

These symmetric modes have a scalar and a vector field contribution. Hence the total electron-phonon coupling Hamiltonian can be written as

$$H_{\text{e-ph}} = H^A_{\text{e-ph}} + H^S_{\text{e-ph}} \tag{9}$$

At small angles the gauge phonon contribution comes mainly from $H^A_{\text{e-ph}}$ since $\gamma \sim 1/\theta \gg 1$. Hence, gauge phonons become the dominant mechanism for transport at low angles [9].In monolayer graphene the deformation potential constant is and order of magnitude higher than the gauge field coupling constant, however in tBG, because of large screening effects and no enhancement at small angles the deformation potential contribution becomes irrelevant.

We see the dominance of the geometrically enhanced antisymmetric gauge phonon modes clearly in Supplementary Fig. 7 where we plot the electron-phonon scattering rates for the antisymmetric gauge phonon contribution, the gauge contribution of the symmetric phonon mode and the dynamically screened deformation potential contribution of the symmetric phonon mode. At large angles the first two contributions are similar, however at smaller angles, especially close to the magic angle, because $\gamma \sim 1/\theta \gg 1$, the antisymmetric gauge phonon mode dominates transport. The bare (unscreened) deformation potential has no fermi velocity renormalization effect neither does it have a geometric enhancement and hence is similar to that of monolayer graphene ($D'_A \approx D_A$) [5, 11]. Moreover, due to the large screening effect, the deformation potential contribution is irrelevant at all angles. We note that the validity of these arguments hold only when $\tilde{a} \ll L$, where L is the dimension of the sample, and $\tilde{a} = a_0/[2\sin(\theta/2)]$ is the lattice vector of the moiré superlattice, where $a_0 = 2.46$ Å is the graphene lattice constant. For L of the order of microns, we must have $\theta \gg 0.02^\circ$ for the formalism to be valid. We therefore expect that the divergence of $\tilde{\beta}_A$ as $\theta \to 0$ predicted in this model is unphysical when the moiré period becomes comparable to the sample size.

IV. BOLTZMANN TRANSPORT

A. General formalism

Carrier current is created by applying an electric field $\mathbf{E}$, which has the effect of changing the electronic distribution $f_{\mathbf{k},\lambda}$ from the Fermi-Dirac distribution $f^0_{\mathbf{k}\lambda}$. Up to linear order in response, the distribution $f_{\mathbf{k}\lambda}$ can be expressed as $f_{\mathbf{k},\lambda} = f^0_{\mathbf{k},\lambda} + h_{\mathbf{k},\lambda}$. The change in the distribution function $h_{\mathbf{k},\lambda}$ can be evaluated within the Boltzmann transport formalism. The change in the distribution function due to the electric field is compensated by the collision integral $\mathrm{St}[f_{\mathbf{k},\lambda}]$, which describes the rate of change in the occupation of the electronic states due to scattering.

$$-\frac{e\mathbf{E}}{\hbar}\nabla_{\mathbf{k}} f_{\mathbf{k},\lambda} = \mathrm{St}[f_{\mathbf{k},\lambda}] \tag{10}$$

The collision integral can be written as

$$\mathrm{St}\left[f_{\mathbf{k},\lambda}\right] = \sum_{\mathbf{k}',\lambda',\nu} P^{\lambda'\lambda}_{\mathbf{k}'\mathbf{k},\nu} f_{\mathbf{k}',\lambda'}(1-f_{\mathbf{k},\lambda}) - P^{\lambda\lambda'}_{\mathbf{k}\mathbf{k}',\nu} f_{\mathbf{k},\lambda}(1-f_{\mathbf{k}',\lambda'}), \tag{11}$$

where $P^{\lambda'\lambda}_{\mathbf{k}'\mathbf{k},\nu}$ is the scattering probability from state $|\lambda',\mathbf{k}'\rangle$ to $|\lambda,\mathbf{k}\rangle$ within phonon branch ν (TA or LA), which is given by

$$\begin{aligned} P^{\lambda\lambda'}_{\mathbf{k},\mathbf{k}+\mathbf{q},\nu} = \frac{2\pi}{\hbar}\left|g^{\lambda,\lambda'}_{\mathbf{k},\mathbf{k}+\mathbf{q},\nu}\right|^2 & \left[n_{\mathbf{q},\nu}\delta(\varepsilon_{\mathbf{k}+\mathbf{q},\lambda'} - \varepsilon_{\mathbf{k},\lambda} - \hbar\omega_{\mathbf{q},\nu})\right. \\ & \left. +(1+n_{\mathbf{q},\nu})\delta(\varepsilon_{\mathbf{k}+\mathbf{q},\lambda'} - \varepsilon_{\mathbf{k},\lambda} + \hbar\omega_{\mathbf{q},\nu})\right], \end{aligned} \tag{12}$$

where the two terms account for absorption and emission of phonons, $n_{\mathbf{q},\nu}$ is the Bose-Einstein distribution function describing the phonon population, $\hbar\omega_{\mathbf{q},\nu} = \hbar c_\nu q$ is the phonon energy, and $g^{\lambda,\lambda'}_{\mathbf{k},\mathbf{k}+\mathbf{q},\nu}$ is the electron-phonon coupling, which can be expressed as

$$g^{\lambda,\lambda'}_{\mathbf{k},\mathbf{k}+\mathbf{q},\nu} = \sqrt{\frac{\hbar}{2A\mu_s\omega_{\mathbf{q},\nu}}} M^{\lambda,\lambda'}_{\mathbf{k},\mathbf{k}+\mathbf{q}} \tag{13}$$

where A is the area of the graphene layer, μ_s is the mass density, and $M^{\lambda,\lambda'}_{\mathbf{k},\mathbf{k}+\mathbf{q}}$ is the matrix element for scattering between initial and final states, which is given by

$$M^{\lambda,\lambda'}_{\mathbf{k},\mathbf{k}+\mathbf{q}} = \zeta q\left[F^{\lambda\lambda'}_{\mathbf{k},\mathbf{k}+\mathbf{q}}\right]^{1/2}, \tag{14}$$

where ζ is the effective deformation potential and $F^{\lambda\lambda'}_{\mathbf{k},\mathbf{k}+\mathbf{q}}$ is the tBG chirality factor, which for Dirac model is given by $(1+\lambda\lambda'\cos\theta_{\mathbf{k},\mathbf{k}+\mathbf{q}})/2$. The effective deformation potential ζ stands for either the effective scalar potential $\tilde{D}_A$ or twice the effective gauge potential $2\tilde{\beta}_A$ (see Appendix. III). Using the detailed balance condition

$$P^{\lambda'\lambda}_{\mathbf{k}'\mathbf{k}} f^0_{\mathbf{k}',\lambda'}(1-f^0_{\mathbf{k},\lambda}) = P^{\lambda\lambda'}_{\mathbf{k}\mathbf{k}'} f^0_{\mathbf{k},\lambda}(1-f^0_{\mathbf{k}',\lambda'}), \tag{15}$$

one can verify that the the ansatz

$$h_{\mathbf{k},\lambda} = \lambda eE\tau_{\mathbf{k},\lambda}\cos\theta_{\mathbf{k}} v_F \frac{\partial f^0_{\mathbf{k},\lambda}}{\partial\varepsilon_{\mathbf{k},\lambda}} \tag{16}$$

solves the Boltzmann equation (Supplementary Eq. 10), where $\varepsilon_{\mathbf{k},\lambda}$ is the energy dispersion. Furthermore, we assume $\tau_{\mathbf{k}',\lambda'} \approx \tau_{\mathbf{k},\lambda}$, which is a reasonable assumption for impurity assisted electron-phonon scattering [5]. The scattering time $\tau_{\mathbf{k},\lambda}$ can be solved for as

$$\frac{1}{\tau_{\mathbf{k},\lambda}} = \sum_{\substack{\lambda',\mathbf{k}' \\ \nu=\mathrm{LA,TA}}} (1-\lambda\lambda'\cos\theta_{\mathbf{k}\mathbf{k}'})\frac{1-f^0_{\mathbf{k}',\lambda'}}{1-f^0_{\mathbf{k},\lambda}} P^{\lambda\lambda'}_{\mathbf{k}\mathbf{k}',\nu}, \tag{17}$$

where $\theta_{\mathbf{k}\mathbf{k}'}$ is the scattering angle between the initial and final states. The case of $\lambda = \lambda'$ and $\lambda \neq \lambda'$ corresponds to intraband and interband scattering respectively. The momentum dependence of the scattering time can be shown to enter only through the energy dispersion $\epsilon_{\mathbf{k},\lambda} = \lambda\hbar v_F k$ i.e. $\tau^{e-\mathrm{ph}}_{\mathbf{k},\lambda} = \tau^{e-\mathrm{ph}}(\varepsilon_{\mathbf{k},\lambda})$.

Using the fact that

$$n_{\mathbf{q},\nu}\frac{1-f^0_{\mathbf{k}',\lambda'}}{1-f^0_{\mathbf{k},\lambda}} = f^0_{\mathbf{k}',\lambda'} + n_{\mathbf{q},\nu}, \tag{18}$$

$$(1+n_{\mathbf{q},\nu})\frac{1-f^0_{\mathbf{k}',\lambda'}}{1-f^0_{\mathbf{k},\lambda}} = -f^0_{\mathbf{k}',\lambda'} + n_{\mathbf{q},\nu} + 1, \tag{19}$$

we find that the scattering rates take the form

$$\frac{1}{\tau^a_{\mathbf{k},\lambda}} = \sum_{\lambda',\nu=\mathrm{TA,LA}} \int \frac{d\mathbf{q}}{(2\pi)} \frac{|M^{\lambda\lambda'}_{\mathbf{k},\mathbf{k}+\mathbf{q}}|^2}{2\mu_s\omega_{\mathbf{q},\nu}} (1+\lambda\lambda'\cos\theta_{\mathbf{k},\mathbf{k}+\mathbf{q}}) \left(f^0_{\mathbf{k}',\lambda'} + n_{\mathbf{q},\nu}\right)\delta\left(\varepsilon_{\mathbf{k}+\mathbf{q},\lambda'} - \varepsilon_{\mathbf{k},\lambda} - \hbar\omega_{\mathbf{q},\nu}\right) \tag{20}$$

$$\frac{1}{\tau^e_{\mathbf{k},\lambda}} = \sum_{\lambda',\nu=\mathrm{TA,LA}} \int \frac{d\mathbf{q}}{(2\pi)} \frac{|M^{\lambda\lambda'}_{\mathbf{k},\mathbf{k}+\mathbf{q}}|^2}{2\mu_s\omega_{\mathbf{q},\nu}} (1+\lambda\lambda'\cos\theta_{\mathbf{k},\mathbf{k}+\mathbf{q}}) \left(1-f^0_{\mathbf{k}',\lambda'} + n_{\mathbf{q},\nu}\right)\delta\left(\varepsilon_{\mathbf{k}+\mathbf{q},\lambda'} - \varepsilon_{\mathbf{k},\lambda} + \hbar\omega_{\mathbf{q},\nu}\right), \tag{21}$$

where the subscript a (e) indicate phonon absorption (emission) respectively and the total rate is the sum of the two. The delta functions can be simplified, and the intraband scattering rate becomes

$$\frac{1}{\tau^{\mathrm{intra,(a)}}_{\mathbf{k},\lambda}} = \sum_{\nu=\mathrm{TA,LA}} \frac{\zeta^2}{2\hbar\mu_s c_\nu} \int_0^{2k/(1-\lambda z_\nu)} \frac{dq}{2\pi}\frac{q^3}{k} \frac{\sqrt{1-s^2_{\lambda,\nu}}}{\sqrt{k^2+q^2+2kqs_{\lambda,\nu}}} \left\{ f_0\left(\hbar v_F k\left[\lambda + z_\nu\frac{q}{k}\right]\right) + n_{\mathbf{q},\nu}\right\} \tag{22}$$

$$\frac{1}{\tau^{\mathrm{intra,(e)}}_{\mathbf{k},\lambda}} = \sum_{\nu=\mathrm{TA,LA}} \frac{\zeta^2}{2\hbar\mu_s c_\nu} \int_0^{2k/(1+\lambda z_\nu)} \frac{dq}{2\pi}\frac{q^3}{k} \frac{\sqrt{1-s^2_{-\lambda,\nu}}}{\sqrt{k^2+q^2+2kqs_{-\lambda,\nu}}} \left\{1- f_0\left(\hbar v_F k\left[\lambda - z_\nu\frac{q}{k}\right]\right) + n_{\mathbf{q},\nu}\right\}, \tag{23}$$

where $s_{\lambda,\nu} = (q/2k)(z_\nu^2-1)+\lambda z_\nu$, $z_\nu = c_\nu/v_F$, $f_0(x) = 1/\{\exp[(x-\mu)/k_BT]+1\}$.

In the limit of $c_\nu \ll v_F$, the intraband scattering rate can be simplified to

$$\frac{1}{\tau^{\mathrm{intra,(a)}}_{\mathbf{k},\lambda}} \approx \sum_{\nu=\mathrm{TA,LA}} \frac{\zeta^2}{2\hbar\mu_s c_\nu} \int_0^{2k} \frac{dq}{2\pi}\frac{q^3}{k}\sqrt{1-(q/2k)^2}\left(f^0_{\mathbf{k},\lambda} + n_{\mathbf{q},\nu}\right) \tag{24}$$

$$\frac{1}{\tau^{\mathrm{intra,(e)}}_{\mathbf{k},\lambda}} \approx \sum_{\nu=\mathrm{TA,LA}} \frac{\zeta^2}{2\hbar\mu_s c_\nu} \int_0^{2k} \frac{dq}{2\pi}\frac{q^3}{k}\sqrt{1-(q/2k)^2}\left(1-f^0_{\mathbf{k},\lambda} + n_{\mathbf{q},\nu}\right), \tag{25}$$

The total intraband scattering rate in the limit of $c_\nu \ll v_F$ is the sum of the scattering rate due to absorption and emission

$$\frac{1}{\tau^{\mathrm{intra}}_{\mathbf{k},\lambda}} \approx \sum_{\nu=\mathrm{TA,LA}} \frac{\zeta^2}{2\hbar\mu_s c_\nu} \int_0^{2k} \frac{dq}{2\pi}\frac{q^3}{k}\sqrt{1-(q/2k)^2}\,(1+2n_{\mathbf{q},\nu}). \tag{26}$$

The interband scattering rates is given by

$$\frac{1}{\tau^{\mathrm{inter,(a)}}_{\mathbf{k},\lambda=-1}} = \sum_{\nu=\mathrm{TA,LA}} \frac{\zeta^2}{2\hbar\mu_s c_\nu} \int_{2k/(z_\nu+1)}^{2k/(z_\nu-1)} \frac{dq}{2\pi}\frac{q^3}{k} \frac{\sqrt{1-s^2_{-,\nu}}}{\sqrt{k^2+q^2+2kqs_{-,\nu}}} \left\{ f_0\left(\hbar v_F k\left[z_\nu\frac{q}{k} - 1\right]\right) + n_{\mathbf{q},\nu}\right\} \tag{27}$$

$$\frac{1}{\tau^{\mathrm{inter,(e)}}_{\mathbf{k},\lambda=1}} = \sum_{\nu=\mathrm{TA,LA}} \frac{\zeta^2}{2\hbar\mu_s c_\nu} \int_{2k/(z_\nu+1)}^{2k/(z_\nu-1)} \frac{dq}{2\pi}\frac{q^3}{k} \frac{\sqrt{1-s^2_{-,\nu}}}{\sqrt{k^2+q^2+2kqs_{-,\nu}}} \left\{1- f_0\left(\hbar v_F k\left[1 - z_\nu\frac{q}{k}\right]\right) + n_{\mathbf{q},\nu}\right\}, \tag{28}$$

where $s_{-,\nu} = (q/2k)(z_\nu^2-1) - z_\nu$, $z_\nu = c_\nu/v_F$, $f_0(x) = 1/\{\exp[(x-\mu)/k_BT]+1\}$. Note that while the intraband scattering is the sum of both absorption and emission, the interband scattering can only be either of them depending on λ.

The resistivity $\rho_{e-\mathrm{ph}}$ is obtained from the scattering time $\tau^{e-\mathrm{ph}}(\varepsilon)$ by the energy average

$$\frac{1}{\rho_{e-ph}} = e^2\int d\varepsilon N_D(\varepsilon)\frac{v_F^2}{2}\tau^{e-\mathrm{ph}}(\varepsilon)\left(-\frac{\partial f^0(\varepsilon)}{\partial\varepsilon}\right), \tag{29}$$

where N_D is density of states.

B. Beyond the Dirac approximation

In this subsection, we provide a detailed explanation of the effective model in Eq. 2 of the main text including density of states (DOS), scattering time and resistivity. Without loss of generality, we locate K and K' points along k_y axis, i.e. $\Delta K = ik_\theta$ Hence

$$H = -\frac{\hbar v_F}{k_\theta}\begin{pmatrix} 0 & k^{*2} - (-ik_\theta/2)^2 \\ k^2 - (ik_\theta/2)^2 & 0 \end{pmatrix}, \tag{30}$$

where $k = k_x + ik_y$. Introducing $\varepsilon_{\rm VHS} = (1/4)\hbar v_F k_\theta$, which corresponds to energy at van Hove singularity, we can write the Hamiltonian as

$$H = -\varepsilon_{\rm VHS}\begin{pmatrix} 0 & 4\left(\tilde{k}_x - i\tilde{k}_y\right)^2 + 1 \\ 4\left(\tilde{k}_x + i\tilde{k}_y\right)^2 + 1 & 0 \end{pmatrix}, \tag{31}$$

where $\tilde{\mathbf{k}} = \mathbf{k}/k_\theta$.

To circumvent difficulties with anisotropic dispersion, we map this Hamiltonian to massless Dirac Hamiltonian

$$H = \varepsilon_{\rm VHS} r\begin{pmatrix} 0 & e^{-i\phi} \\ e^{i\phi} & 0 \end{pmatrix} \tag{32}$$

where $\varepsilon_{\rm VHS} = (1/4)\hbar v_F k_\theta$.

This can be achieved by using the following variable transformation from (k_x, k_y) to (r, ϕ)

$$k_x = -(\gamma/2)k_\theta {\rm sgn}\,(\sin\phi)\sqrt{\frac{1}{2}\left[-(1 + r\cos\phi) + \sqrt{(1 + r\cos\phi)^2 + (r\sin\phi)^2}\right]} \tag{33}$$

$$k_y = (\gamma/2)k_\theta\sqrt{\frac{1}{2}\left[1 + r\cos\phi + \sqrt{(1 + r\cos\phi)^2 + (r\sin\phi)^2}\right]}, \tag{34}$$

where $\gamma = \pm 1$ represents each half of the Fermi surface. Note that Fermi surface splitting happens at energy $|E| < \varepsilon_{\rm VHS}$. The expression in Supplementary Eq. 32 resembles that of monolayer graphene and hence we can calculate the resistivity as in monolayer graphene, with the caveat that we also need to introduce the determinant of the Jacobian matrix of the transformation in the integrals.

The range of the new co-ordinate ϕ is $\phi \in [-\pi, \pi]$. In the transformed coordinates, the energy dispersion and the corresponding eigenstates are given by

$$E_\pm(r) = \pm\varepsilon_{\rm VHS} r \tag{35}$$

$$|\pm\rangle = \frac{1}{\sqrt{2}}\begin{pmatrix} \pm 1 \\ e^{i\phi} \end{pmatrix} \tag{36}$$

Note that in the new coordinates, $r = 1$ corresponds to van Hove singularity in the original coordinates. The Jacobian $\mathcal{J}$ corresponding to this transformation is given by

$$\mathcal{J}(r,\phi) = \begin{vmatrix} \frac{\partial k_x}{\partial r} & \frac{\partial k_x}{\partial \phi} \\ \frac{\partial k_y}{\partial r} & \frac{\partial k_y}{\partial \phi} \end{vmatrix} = \frac{k_\theta^2 r}{16\sqrt{(1 + r\cos\phi)^2 + (r\sin\phi)^2}} \tag{37}$$

The density of states at energy below the Van Hove singularity $|\varepsilon| < \varepsilon_{\rm VHS}$ can be obtained analytically as

$$\begin{aligned} D(\varepsilon) &= \frac{g}{2}\int \frac{d^2k}{(2\pi)^2}\delta(\varepsilon - \varepsilon_{\mathbf{k},+}) \\ &= \frac{g}{2}\sum_{\gamma=\pm 1}\int_0^\infty dr\int_{-\pi}^{\pi} d\phi \frac{\mathcal{J}(r,\phi)}{(2\pi)^2}\delta(\varepsilon - \varepsilon_{\rm VHS} r) \\ &= \frac{1}{2}\left(\frac{n_{\rm VHS}}{\varepsilon_{\rm VHS}}\right)\left(\frac{|\varepsilon|}{\varepsilon_{\rm VHS}}\right)\sum_{z=\pm 1}\frac{K\left[4z\frac{|\varepsilon|/\varepsilon_{\rm VHS}}{(1+z|\varepsilon|/\varepsilon_{\rm VHS})^2}\right]}{1 + z\left(|\varepsilon|/\varepsilon_{\rm VHS}\right)}, \end{aligned} \tag{38}$$

where $g = 8$ is the degeneracy, $K(x) = (\pi/2)\sum_{n=0}^{\infty}[(2n-1)!!/(2n)!!]^2 x^{2n}$ is the complete elliptic integral of the first kind, and $n_{\rm VHS}$ is the density at VHS, given by $n_{\rm VHS} = [g/(16\pi^2)]k_\theta^2$.

In the following paragraph, we provide a detailed calculation of the scattering time from the effective model in described above. The electron-phonon scattering time can be obtained from the standard Boltzmann formula

$$\frac{1}{\tau^{(j)}_{\mathbf{k},\lambda}} = \sum_{\substack{\lambda',\mathbf{k}'\\ \nu=\mathrm{LA,TA}}} P^{\lambda,\lambda'}_{\mathbf{k},\mathbf{k}',\nu}\frac{1-f^0_{\mathbf{k}',\lambda'}}{1-f^0_{\mathbf{k},\lambda}}\left(1-\frac{v^{(j)}_{\mathbf{k}',\lambda'}}{v^{(j)}_{\mathbf{k},\lambda}}\frac{\tau^{(j)}_{\mathbf{k}',\lambda'}}{\tau^{(j)}_{\mathbf{k},\lambda}}\right)$$
$$= A\sum_{\lambda',\nu=\mathrm{TA,LA}}\int\frac{d^2\mathbf{k}'}{(2\pi)^2}P^{\lambda,\lambda'}_{\mathbf{k},\mathbf{k}',\nu}\frac{1-f^0_{\mathbf{k}',\lambda'}}{1-f^0_{\mathbf{k},\lambda}}\left(1-\frac{v^{(j)}_{\mathbf{k}',\lambda'}}{v^{(j)}_{\mathbf{k},\lambda}}\frac{\tau^{(j)}_{\mathbf{k}',\lambda'}}{\tau^{(j)}_{\mathbf{k},\lambda}}\right) \tag{39}$$

which in the new coordinates $\mathbf{p}' = (r', \phi')$ can be written as

$$\frac{1}{\tau^{(j)}_{\mathbf{k},\lambda,\gamma}} = A\sum_{\gamma',\lambda',\nu}\int_0^\infty dr'\int_{-\pi}^{\pi}d\phi'\frac{\mathcal{J}(r',\phi')}{(2\pi)^2}P^{\lambda,\lambda';\gamma,\gamma'}_{\mathbf{k},\mathbf{k}',\nu}\frac{1-f^0_{\mathbf{k}',\lambda'}}{1-f^0_{\mathbf{k},\lambda}}\left(1-\frac{v^{(j)}_{\mathbf{k}',\lambda',\gamma'}}{v^{(j)}_{\mathbf{k},\lambda,\gamma}}\frac{\tau^{(j)}_{\mathbf{k}',\lambda',\gamma'}}{\tau^{(j)}_{\mathbf{k},\lambda,\gamma}}\right) \tag{40}$$

We include only electron-phonon intravalley scattering, i.e.

$$P^{\lambda,\lambda';\gamma,\gamma'}_{\mathbf{k},\mathbf{k}',\nu} = \begin{cases} P^{\lambda,\lambda'}_{\mathbf{k},\mathbf{k}',\nu} & ;\gamma'=\gamma \\ 0 & ;\gamma'\neq\gamma\end{cases}, \tag{41}$$

and we make the following approximation

$$1-\frac{v^{(j)}_{\mathbf{k}',\lambda'}}{v^{(j)}_{\mathbf{k},\lambda}}\frac{\tau_{\mathbf{k}',\lambda'}}{\tau_{\mathbf{k},\lambda}} \to 1-\cos\phi'_{\mathbf{p},\mathbf{p}'}, \tag{42}$$

which have negligible effect in resistivity calculation.

Hence, we can express the scattering rate as

$$\frac{1}{\tau} \approx A\sum_{\substack{\lambda'\\ \nu=\mathrm{TA,LA}}}\int_0^\infty dr'\int_{-\pi}^{\pi}d\phi'\frac{\mathcal{J}(r',\phi')}{(2\pi)^2}P^{\lambda,\lambda'}_{\mathbf{k},\mathbf{k}',\nu}\frac{1-f^0_{\mathbf{k}',\lambda'}}{1-f^0_{\mathbf{k},\lambda}}\left(1-\cos\phi'_{\mathbf{p},\mathbf{p}'}\right) \tag{43}$$

Now we study the separate contributions from intraband and interband scattering.

Intraband $(v_F > c)$ *:*

The intraband scattering rate is

$$\frac{1}{\tau^{\rm intra}_{r,\phi,\lambda}} = \frac{1}{\tau^{\rm intra,(a)}_{r,\phi,\lambda}} + \frac{1}{\tau^{\rm intra,(e)}_{r,\phi,\lambda}} \tag{44}$$

$$\frac{1}{\tau^{\rm intra,(a)}_{r,\phi,\lambda}} = \sum_{\nu=\mathrm{TA,LA}}\frac{16\pi\tilde{\beta}_A^2}{\mu_s\hbar c_\nu v_F k_\theta}\int_0^\infty dr'\int_{-\pi}^{\pi}d\phi'\frac{\mathcal{J}(r',\phi')}{(2\pi)^2}q(r,\phi,r',\phi')F^+_{\mathbf{p},\mathbf{p}'}\left[1-\cos\left(\phi'-\phi\right)\right]\left(f^0_{\mathbf{p}',\lambda}+n_{\mathbf{q},\nu}\right)$$
$$\times\,\delta\left(\lambda(r'-r)-zr\frac{q(r,\phi,r',\phi')}{p}\right) \tag{45}$$

$$\frac{1}{\tau^{\rm intra,(e)}_{r,\phi,\lambda}} = \sum_{\nu=\mathrm{TA,LA}}\frac{16\pi\tilde{\beta}_A^2}{\mu_s\hbar c_\nu v_F k_\theta}\int_0^\infty dr'\int_{-\pi}^{\pi}d\phi'\frac{\mathcal{J}(r',\phi')}{(2\pi)^2}q(r,\phi,r',\phi')F^+_{\mathbf{p},\mathbf{p}'}\left[1-\cos\left(\phi'-\phi\right)\right]\left(1-f^0_{\mathbf{p}',\lambda}+n_{\mathbf{q},\nu}\right)$$
$$\times\,\delta\left(\lambda(r'-r)+zr\frac{q(r,\phi,r',\phi')}{p}\right), \tag{46}$$

where $z_\nu = c_\nu/v_F$, $p = (r/4)k_\theta$, $F^+_{\mathbf{p},\mathbf{p}'} = [1+\cos(\phi'-\phi)]/2$ is chirality of tBG in transformed coordinates, and $q(r,\phi,r',\phi')$ is momentum transferred by phonon, which explicitly is given by

$$q(r,\phi,r',\phi') = \sqrt{\left[k'_x\left(r',\phi'\right)-k_x\left(r,\phi\right)\right]^2+\left[k'_y\left(r',\phi'\right)-k_y\left(r,\phi\right)\right]^2}, \tag{47}$$

(see Supplementary Eq. 34 for variable transformation from (k_x, k_y) to (r, ϕ)). Combining both scattering terms for absorption and emission leads to Eq. 3 in the main text.

Interband ($v_F < c$) *:*

Similarly for interband we get the following expression for scattering rate.

$$\frac{1}{\tau^{\text{inter,(a)}}_{r,\phi,\lambda=-1}} = \frac{16\pi\tilde{\beta}_A^2}{\mu_s \hbar c_\nu v_F k_\theta} \sum_{\nu=\text{TA,LA}} \int_0^\infty dr' \int_{-\pi}^{\pi} d\phi' \frac{\mathcal{J}(r',\phi')}{(2\pi)^2} q(r,\phi,r',\phi') F^{-}_{\mathbf{p},\mathbf{p}'} \left[1+\cos\left(\phi'-\phi\right)\right] \left(f^0_{\mathbf{p}',+1} + n_{\mathbf{q},\nu}\right) \times \delta\left(r' + r - zr\frac{q(r,\phi,r',\phi')}{p}\right) \tag{48}$$

$$\frac{1}{\tau^{\text{inter,(e)}}_{r,\phi,\lambda=1}} = \frac{16\pi\tilde{\beta}_A^2}{\mu_s \hbar c_\nu v_F k_\theta} \sum_{\nu=\text{TA,LA}} \int_0^\infty dr' \int_{-\pi}^{\pi} d\phi' \frac{\mathcal{J}(r',\phi')}{(2\pi)^2} q(r,\phi,r',\phi') F^{-}_{\mathbf{p},\mathbf{p}'} \left[1+\cos\left(\phi'-\phi\right)\right] \left(1 - f^0_{\mathbf{p}',-1} + n_{\mathbf{q},\nu}\right) \times \delta\left(-(r'+r) + zr\frac{q(r,\phi,r',\phi')}{p}\right), \tag{49}$$

where $z_\nu = c_\nu/v_F$, $p = (r/4)k_\theta$, $q(r,\phi,r',\phi')$ is momentum transferred by phonon (see Supplementary Eq. 47), and $F^{-}_{\mathbf{p},\mathbf{p}'} = [1-\cos(\phi'-\phi)]/2$ is chirality of tBG in transformed coordinates. Just like the Dirac model, the interband scattering for this two-band effective model can only be either absorption or emission depending on λ. Simplification leads to Eq. 4 in the main text.

Numerical implementation

In order to compute the scattering time, we need to self-consistently solve for r' (r,ϕ,ϕ') before resolving the delta function. Special care is needed to capture possible multiple solution of r' for some regime of r, ϕ, and ϕ'. Alternatively, one might also choose compute it without resolving the delta function. This can be achieved with broadening the delta function into a lorentzian with a very small width, and performing the integration using efficient grid in (r',ϕ') plane.

An efficient grid has to have denser meshes near the region where the argument of delta function vanishes, i.e. the region where scattering takes place. For each (r,ϕ), this can be obtained by sampling of momentum space (r',ϕ') using self-adaptive meshes. Similar technique have been used for different purpose (see. [14] for example). For completeness, we outline the procedure below. First, we define a dimensionless energy scale ξ and make an estimate of integration bound which encompasses the scattering phase space. The integration bound for ϕ' can easily be taken from 0 to 2π, while the integration bound for r' is taken from 0 up to a maximum value r'_{max}. This r'_{max} does not need to be equal to the largest r' in the scattering phase space which obey the conservation of energy (which we don't know anyway). But rather, it can be larger than that, since the lorentzian will act as a cutoff. This integration bound corresponds to rectangular region in the scattering phase space. We then divide this rectangle into four smaller equal-area rectangles. We further break a specific rectangle into four smaller equal-area ones if any $|\arg(r,\phi,r',\phi')| \leq \xi$, where the momentum (r',ϕ') correspond to the center of that specific rectangle and $\arg(r,\phi,r',\phi')$ is the argument of the delta function. We then lower the energy scale as $\xi \to \xi/a$, where $a > 1$, and repeat the above process recursively until desired mesh density is achieved. Note that in general, r'_{max} would be different for each (r,ϕ).

Resistivity

Electron-phonon resistivity. Finally, the resistivity is given by

$$\frac{1}{\rho_{ij}} = e^2 \frac{g}{2} \sum_{\lambda=\pm1} \int \frac{d^2k}{(2\pi)^2} v^{(i)}_{\mathbf{k},\lambda} v^{(j)}_{\mathbf{k},\lambda} \tau^{e-\text{ph}}_{\mathbf{k},\lambda} \left(-\frac{\partial f^0(\varepsilon)}{\partial\varepsilon}\right) \tag{50}$$

$$= e^2 g \sum_{\lambda=\pm1} \int_0^\infty dr \int_{-\pi}^{\pi} d\phi \frac{\mathcal{J}(r,\phi)}{(2\pi)^2} v^{(i)}(r,\phi) v^{(j)}(r,\phi) \tau^{e-\text{ph}}(r,\phi) \left(-\frac{\partial f^0(\varepsilon)}{\partial\varepsilon}\right), \tag{51}$$

where $g = 8$ (degeneracy) and $v^{(j)}_{\mathbf{k},\lambda}$ is band velocity in j direction.

The band velocity $v_{\mathbf{k},\lambda}^{(j)} = (1/\hbar)(\partial\varepsilon_{\mathbf{k},\lambda}/\partial k_j)$ in this anisotropic model in x and y direction is given by

$$v_{\mathbf{k},\lambda}^{(x)} = -\lambda\gamma v_F \mathrm{sgn}\,(\sin\phi)\,\frac{u(r,\phi)+1}{r}\sqrt{\frac{u(r,\phi)-(1+r\cos\phi)}{2}} \tag{52}$$

$$v_{\mathbf{k},\lambda}^{(y)} = \lambda\gamma v_F \frac{u(r,\phi)-1}{r}\sqrt{\frac{u(r,\phi)+1+r\cos\phi}{2}}, \tag{53}$$

where $u(r,\phi) \equiv \sqrt{(1+r\cos\phi)^2+(r\sin\phi)^2}$.

Planckian resistivity. Using Planckian scattering rate $\hbar\tau_{\mathrm{Pl}}^{-1} = Ck_BT$ and introducing dimensionless variables $\tilde{\mathcal{J}} = \mathcal{J}/k_\theta^2$, $\tilde{\mu} = \mu/k_BT$, and $\tilde{v}_{\mathbf{k},\lambda}^{(j)} = \partial\tilde{\varepsilon}_{\tilde{\mathbf{k}},\lambda}/\partial\tilde{k}_j$, where $\tilde{\varepsilon}_{\tilde{\mathbf{k}},\lambda} = \varepsilon_{\mathbf{k},\lambda}/\varepsilon_{\mathrm{VHS}}$ and $\tilde{\mathbf{k}} = \mathbf{k}/k_\theta$, it can be simplified into

$$\begin{aligned}\frac{1}{\rho_{\mathrm{Pl}}^{ij}} &= \frac{e^2}{h}\frac{1}{C}\left(\frac{\varepsilon_{\mathrm{VHS}}}{k_BT}\right)^2 \\ &\quad\times\frac{1}{\pi}\sum_{\lambda=\pm1}\int_0^\infty dr\int_{-\pi}^{\pi} d\phi\,\frac{\tilde{\mathcal{J}}(r,\phi)\tilde{v}_{\mathbf{k},\lambda}^{(i)}\tilde{v}_{\mathbf{k},\lambda}^{(j)}}{\cosh^2\left[\frac{1}{2}\left(\lambda r\frac{\varepsilon_{\mathrm{VHS}}}{k_BT}-\tilde{\mu}\right)\right]} \\ &= \frac{e^2}{h}\frac{1}{C}K_j\left(\frac{n}{n_{\mathrm{VHS}}},\frac{k_BT}{\varepsilon_{\mathrm{VHS}}}\right),\end{aligned} \tag{54}$$

where the function K_j is computed numerically.

V. EXTENDED COMPARISON WITH EXPERIMENTS

In this section, we show the corresponding fit for all samples that appear in Fig. 5 of the main manuscript.

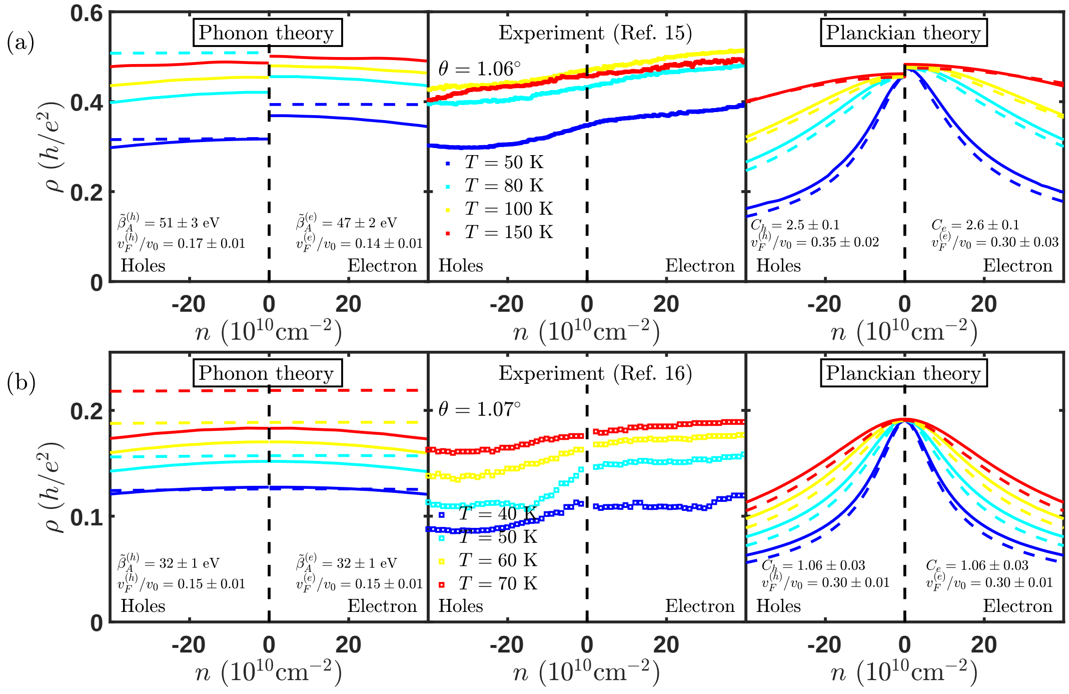

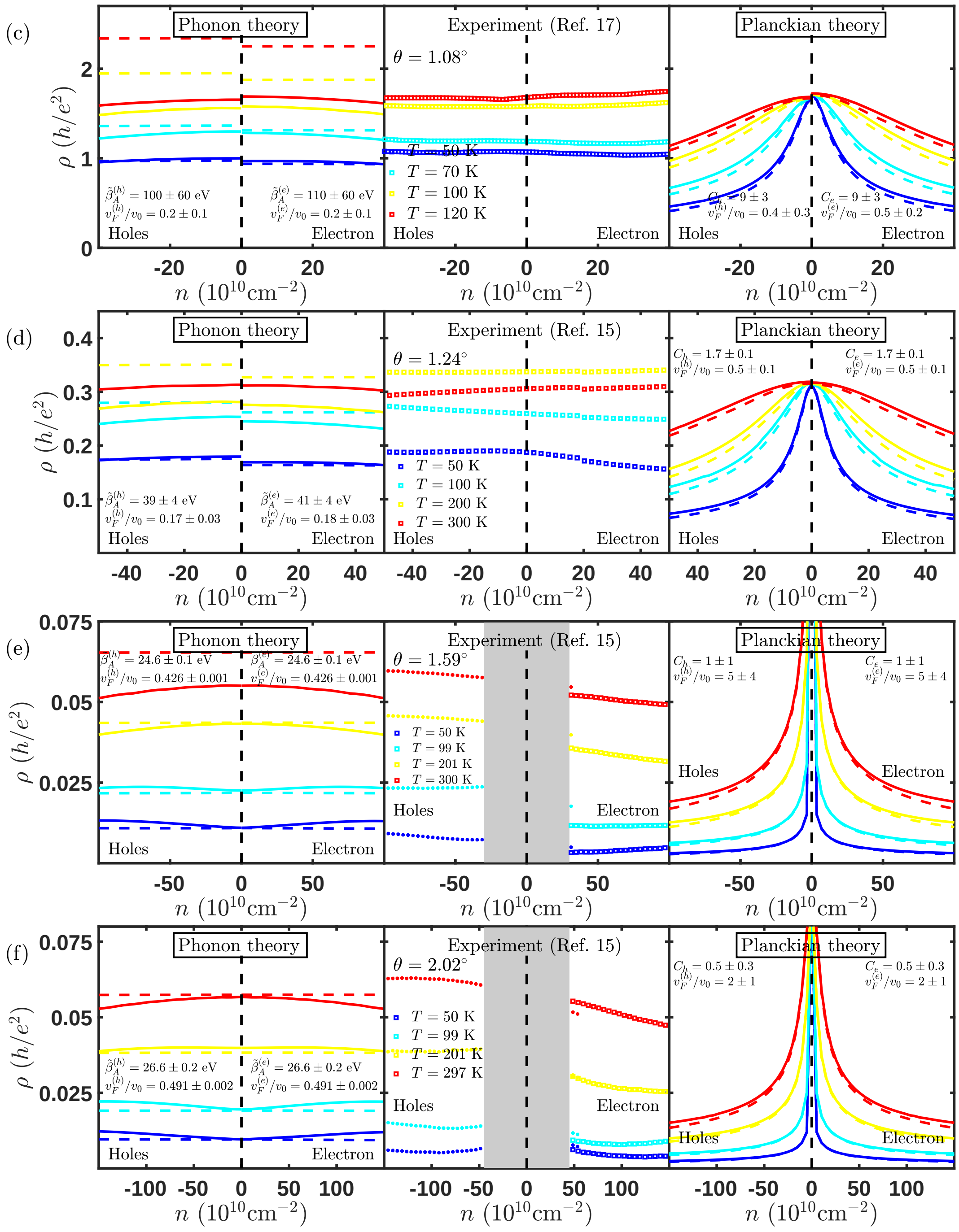

Supplementary Figure 8. Resistivity vs charge density of experiment (middle panel) compared to electron-phonon theory (left panel) and Planckian theory (right panel) at twist angle of (a) 1.06° (Ref. [15]) (b) 1.07° (Ref. [16]) (c) 1.08° (Ref. [17]) (d) 1.24° (Ref. [15]) (e) 1.59° (Ref. [15]) and (f) 2.02° (Ref. [15]). In panels (e) and (f) the low density region is impurity-dominated and has been removed from the analysis (see text for details). The data for for twist angle 1.11° (Ref. [15]) is excluded here since it was already shown in Fig. 2a of the main manuscript.

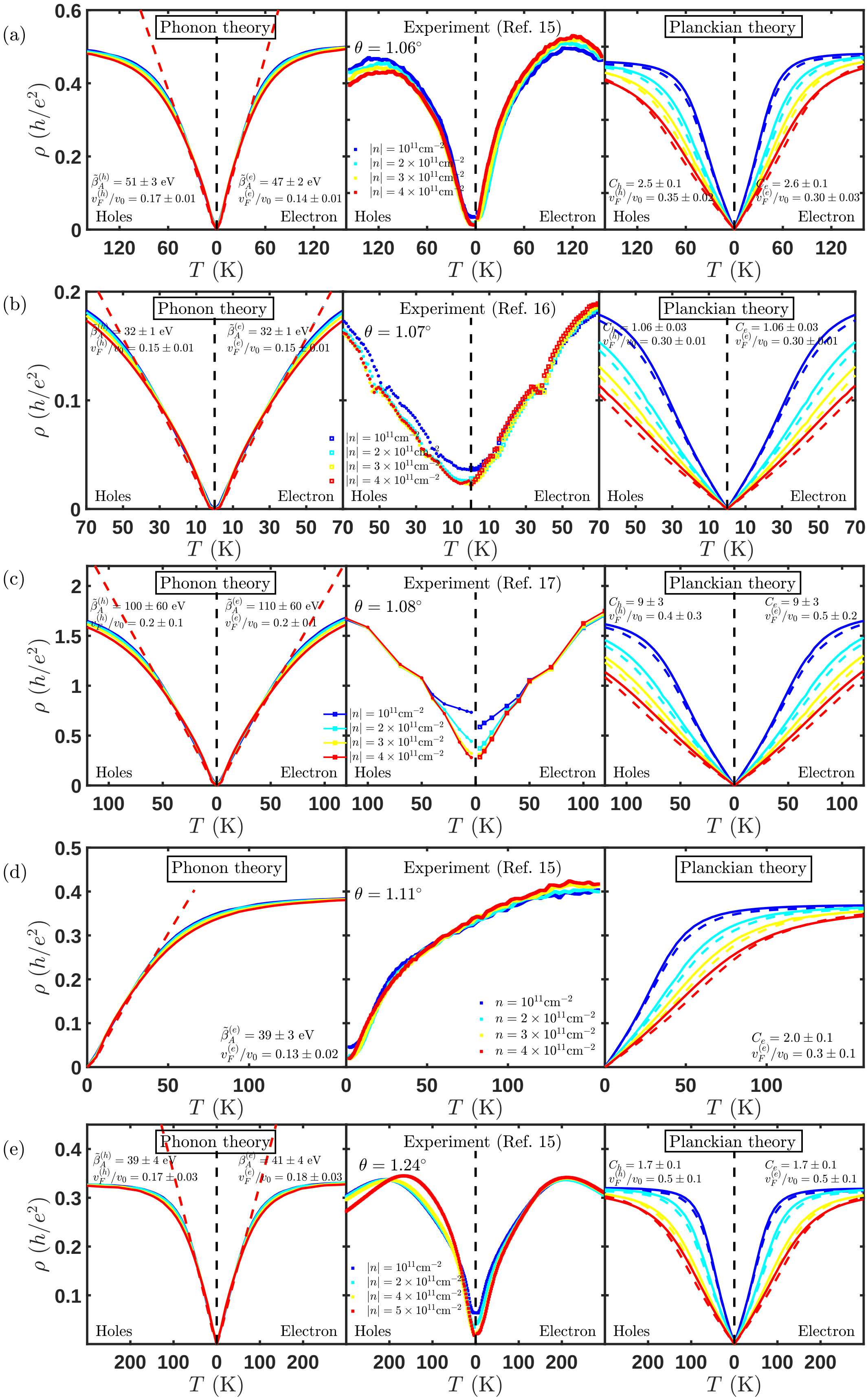
(a)
Phonon theory
Experiment (Ref. 15)
Planckian theory
$\theta = 1.06°$
$\tilde{\beta}_A^{(h)} = 51 \pm 3$ eV
$v_F^{(h)}/v_0 = 0.17 \pm 0.01$
$\tilde{\beta}_A^{(e)} = 47 \pm 2$ eV
$v_F^{(e)}/v_0 = 0.14 \pm 0.01$
$|n| = 10^{11}$cm^{-2}
$|n| = 2 \times 10^{11}$cm^{-2}
$|n| = 3 \times 10^{11}$cm^{-2}
$|n| = 4 \times 10^{11}$cm^{-2}
$C_h = 2.5 \pm 0.1$
$v_F^{(h)}/v_0 = 0.35 \pm 0.02$
$C_e = 2.6 \pm 0.1$
$v_F^{(e)}/v_0 = 0.30 \pm 0.03$
Holes
Electron
ρ (h/e^2)
T (K)
(b)
Phonon theory
Experiment (Ref. 16)
Planckian theory
$\theta = 1.07°$
$\tilde{\beta}_A^{(h)} = 32 \pm 1$ eV
$v_F^{(h)}/v_0 = 0.15 \pm 0.01$
$\tilde{\beta}_A^{(e)} = 32 \pm 1$ eV
$v_F^{(e)}/v_0 = 0.15 \pm 0.01$
$C_h = 1.06 \pm 0.03$
$v_F^{(h)}/v_0 = 0.30 \pm 0.01$
$C_e = 1.06 \pm 0.03$
$v_F^{(e)}/v_0 = 0.30 \pm 0.01$
(c)
Phonon theory
Experiment (Ref. 17)
Planckian theory
$\theta = 1.08°$
$\tilde{\beta}_A^{(h)} = 100 \pm 60$ eV
$v_F^{(h)}/v_0 = 0.2 \pm 0.1$
$\tilde{\beta}_A^{(e)} = 110 \pm 60$ eV
$v_F^{(e)}/v_0 = 0.2 \pm 0.1$
$C_h = 9 \pm 3$
$v_F^{(h)}/v_0 = 0.4 \pm 0.3$
$C_e = 9 \pm 3$
$v_F^{(e)}/v_0 = 0.5 \pm 0.2$
(d)
Phonon theory
Experiment (Ref. 15)
Planckian theory
$\theta = 1.11°$
$\tilde{\beta}_A^{(e)} = 39 \pm 3$ eV
$v_F^{(e)}/v_0 = 0.13 \pm 0.02$
$n = 10^{11}$cm^{-2}
$n = 2 \times 10^{11}$cm^{-2}
$n = 3 \times 10^{11}$cm^{-2}
$n = 4 \times 10^{11}$cm^{-2}
$C_e = 2.0 \pm 0.1$
$v_F^{(e)}/v_0 = 0.3 \pm 0.1$
(e)
Phonon theory
Experiment (Ref. 15)
Planckian theory
$\theta = 1.24°$
$\tilde{\beta}_A^{(h)} = 39 \pm 4$ eV
$v_F^{(h)}/v_0 = 0.17 \pm 0.03$
$\tilde{\beta}_A^{(e)} = 41 \pm 4$ eV
$v_F^{(e)}/v_0 = 0.18 \pm 0.03$
$|n| = 10^{11}$cm^{-2}
$|n| = 2 \times 10^{11}$cm^{-2}
$|n| = 4 \times 10^{11}$cm^{-2}
$|n| = 5 \times 10^{11}$cm^{-2}
$C_h = 1.7 \pm 0.1$
$v_F^{(h)}/v_0 = 0.5 \pm 0.1$
$C_e = 1.7 \pm 0.1$
$v_F^{(e)}/v_0 = 0.5 \pm 0.1$

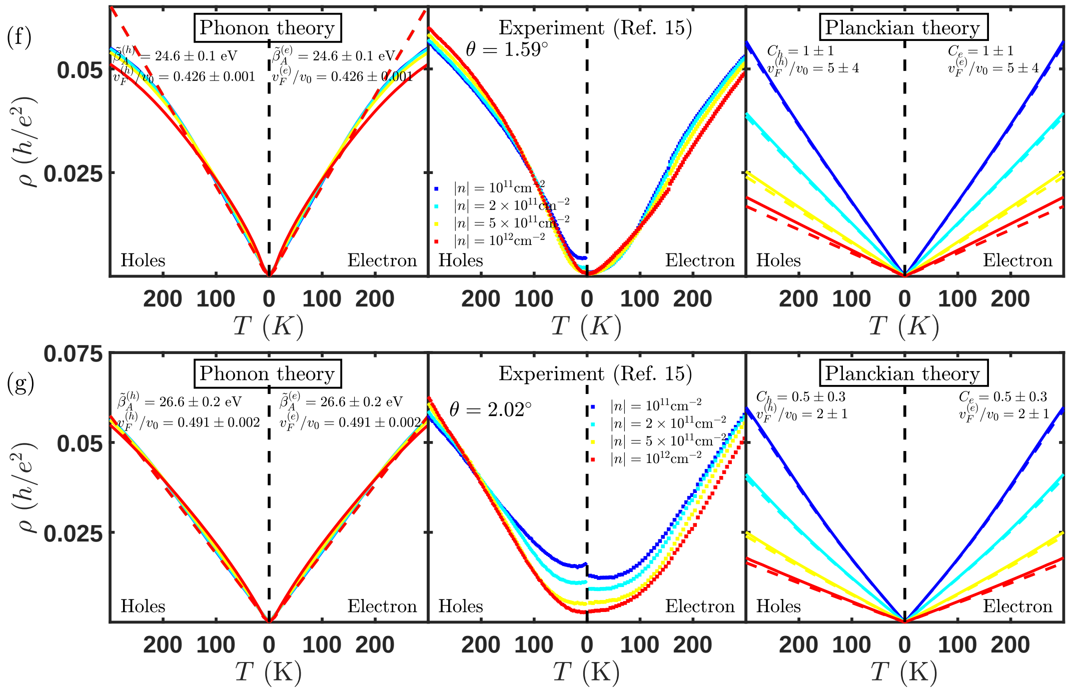

Supplementary Figure 9. Resistivity vs temperature of experiment (middle panel) compared to electron-phonon theory (left panel) and Planckian theory (right panel) at twist angle of (a) 1.06° (Ref. [15]) (b) 1.07° (Ref. [16]) (c) 1.08° (Ref. [17]) (d) 1.11° (Ref. [15]) (e) 1.24° (Ref. [15]) (f) 1.59° (Ref. [15]) and (g) 2.02° (Ref. [15]). The right side of each subplot shows data for electrons, while the left shows data for holes. Panel (d) only shows electrons, since the hole data was already shown in Fig. 2b of the main manuscript.

[1] A. I. Cocemasov, D. L. Nika, and A. A. Balandin, Phys. Rev. B **88**, 035428 (2013).
[2] Y. W. Choi and H. J. Choi, Phys. Rev. B **98**, 241412 (2018).
[3] D. K. Efetov and P. Kim, Phys. Rev. Lett. **105**, 256805 (2010).
[4] J.-H. Chen, C. Jang, S. Xiao, M. Ishigami, and M. S. Fuhrer, Nature nanotechnology **3**, 206 (2008).
[5] T. Sohier, M. Calandra, C.-H. Park, N. Bonini, N. Marzari, and F. Mauri, Phys. Rev. B **90**, 125414 (2014).
[6] S. Ono and K. Sugihara, Journal of the Physical Society of Japan **21**, 861 (1966).
[7] H. Suzuura and T. Ando, Phys. Rev. B **65**, 235412 (2002).
[8] K. Kaasbjerg, K. S. Thygesen, and K. W. Jacobsen, Physical Review B **85**, 165440 (2012).
[9] I. Yudhistira, N. Chakraborty, G. Sharma, D. Y. Ho, E. Laksono, O. P. Sushkov, G. Vignale, and S. Adam, Phys. Rev. B **99**, 140302(R) (2019).
[10] G. Sharma, M. Trushin, O. P. Sushkov, G. Vignale, and S. Adam, Physical Review Research **2**, 022040 (2020).
[11] F. Wu, E. Hwang, and S. Das Sarma, Phys. Rev. B **99**, 165112 (2019).
[12] B. Lian, Z. Wang, and B. A. Bernevig, Phys. Rev. Lett. **122**, 257002 (2019).
[13] M. Koshino and Y.-W. Son, Phys. Rev. B **100**, 075416 (2019).
[14] W.-S. Wang, Y.-Y. Xiang, Q.-H. Wang, F. Wang, F. Yang, and D.-H. Lee, Phys. Rev. B **85**, 035414 (2012).
[15] H. Polshyn, M. Yankowitz, S. Chen, Y. Zhang, K. Watanabe, T. Taniguchi, C. R. Dean, and A. F. Young, Nature Physics **15**, 1011 (2019).
[16] J. M. Park, Y. Cao, K. Watanabe, T. Taniguchi, and P. Jarillo-Herrero, arXiv preprint arXiv:2008.12296 (2020).
[17] Y. Cao, V. Fatemi, A. Demir, S. Fang, S. L. Tomarken, J. Y. Luo, J. D. Sanchez-Yamagishi, K. Watanabe, T. Taniguchi, E. Kaxiras, *et al.*, Nature **556**, 80 (2018).
